# Supplementary material for: ZmNF-YA1 Contributes to Maize Thermotolerance by Regulating Heat Shock Response
Source: Int J Mol Sci. 2024 Jun 6;25(11):6275. doi: 10.3390/ijms25116275 (PMC11173165; doi:10.3390/ijms25116275)
Supplement: Supplementary file 1 [file ijms-25-06275-s001.zip › Supplemental Figures 1-9 (2024=6=5 proof).pdf]

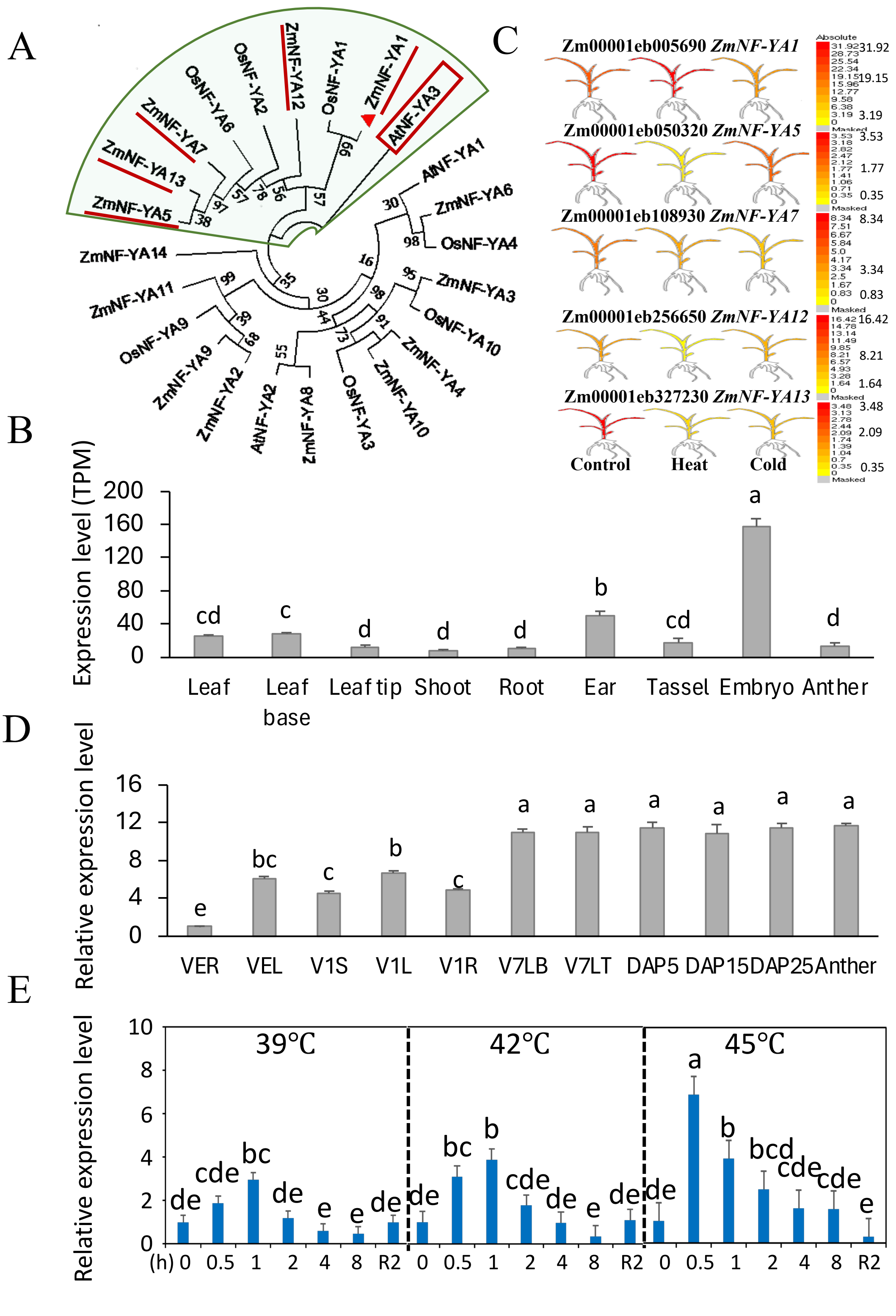

Figure S1 Expression profiles analysis of *ZmNF-YA1*.

(A) Amino acid sequence alignment of ZmNF-YA proteins in maize. The predicted protein sequences of the ZmNF-YA proteins in maize were downloaded from MaizeGDB (https://www.maizegdb.org), with the accession number: *ZmNF-YA1*, Zm00001eb005690; *ZmNF-YA2*, Zm00001eb031690; *ZmNF-YA3*, Zm00001eb032000; *ZmNF-YA4*, Zm00001eb050320; *ZmNF-YA5*, Zm00001d033773; *ZmNF-YA6*, Zm00001eb053830; *ZmNF-YA7*, Zm00001eb108930; *ZmNF-YA8,* Zm00001eb117450; *ZmNF-YA9*, Zm00001eb136080;

ZmNF-YA10, Zm00001eb218610, *ZmNF-YA11*, Zm00001eb216950; *ZmNF-YA12*, Zm00001eb256650; *ZmNF-YA13*, Zm00001eb327230 *ZmNF-YA14*, Zm00001eb430980; *AtNF-YA1*, AT5G12840; *AtNF-YA2*, AT3G05690; *AtNF-YA3*, AT1G72830; *OsNF-YA1*, Os03g0174900; *OsNF-YA2*, Os03g0411100; *OsNF-YA3*, Os03g0647600; *OsNF-YA4*, Os03g0696300; *OsNF-YA6*, Os07g0608200; *OsNF-YA9*, Os12g0613000; *OsNF-YA10*, Os12g0618600. **(B)** Tissue-specific expression profiles of *ZmNF-YA1.* Expression data were downloaded from the MaizeGDB (https://www.maizegdb.org) according to the published works [73-75]. **(C)** Changes in the expression of five NF-YA coding genes in the clade of ZmNF-YA1 response under normal conditions and in response to heat and cold stress treatment Expression data were downloaded from MaizeGDB (https://www.maizegdb.org) according to the published works[73-77]. **(D)** Semi-quantitative RT-PCR and qRT-PCR analysis of *ZmNF-YA1* in different tissues and development stages. VER: The root when the coleoptile is exposed to the ground; VEL: The leaf when the coleoptile is exposed to the ground; V1S: Shoot when the first leaf is fully extended; V1L: Leaf when the first leaf is fully extended; V1R: Root when the first leaf is fully extended; V7LB: The leaf base of the seventh leaf when fully extended; V7LT: Leaf tip of seventh leaf when fully extended; DAP 5/15/25: 5/15/25 days after pollination; Values represent the mean of three replicates ± SD. Different letters denote statistical significance with $P<0.05$ using ANOVA and TukeyHSD test. **(E)** qRT-PCR analysis of *ZmNF-YA1* in response to different heat stress treatments. Seedlings of W22 were grown under a 26 °C/22°C (day/night) temperature regimen at a photon flux density of 700 μmol m^{-2} s^{-1} with a 16 h/8 h light/dark cycle in a chamber with approximately 65% relative humidity for ten days. During HS treatment, half of the plants were transferred to a chamber at a set temperature (39°C, 42°C and 45°C). Approximately 0.1 g of leaf lamina (excluding the midrib) from the middle of the first fully expanded leaf was collected, flash-frozen in liquid nitrogen, and stored in -70°C refrigerator for RNA extraction. Transcript levels were calculated using the $2^{-\Delta\Delta Ct}$ method[49] with maize 18S rRNA[50] as an internal control. The value of *ZmNF-YA1* expression level in WT(W22) before heat stress was set as 1-fold. Values represent the mean of three replicates ± SD ** denotes statistical significance with $P<0.01$ using a *t*-test compared to the value before treatment (0).

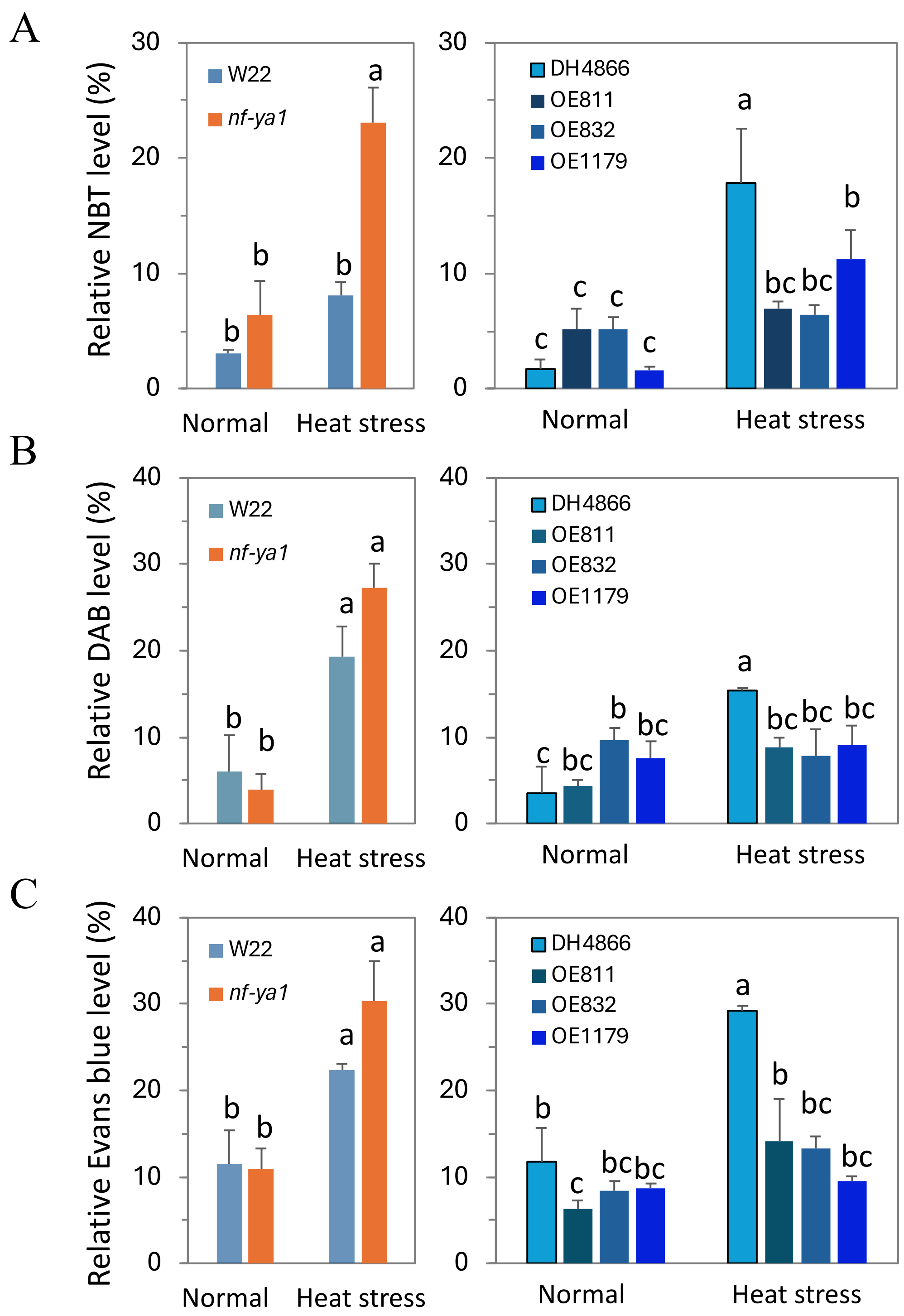

Figure S2 Quantification of NBT, DAB, and Evans blue staining of the **Figure 3D**

Quantification of NBT **(A)**, DAB **(B)**, and Evans blue **(C)** staining of the seedlings from *nf-ya1* mutant, W22, *ZmNF-YA1* OE, and DH4866 lines after heat stress treatment shown in **Figure 3D**. Leaves from 3-leaf stage seedlings before and after 45 °C, 6 h heat stress treatment was collected for ROS staining analysis. Quantification was performed by using analyze particles in ImageJ and then the stanning area (%) was evaluated. Values represent the mean of three replicates ± SD. Different letters denote statistical significance with P<0.05 using ANOVA and TukeyHSD test.

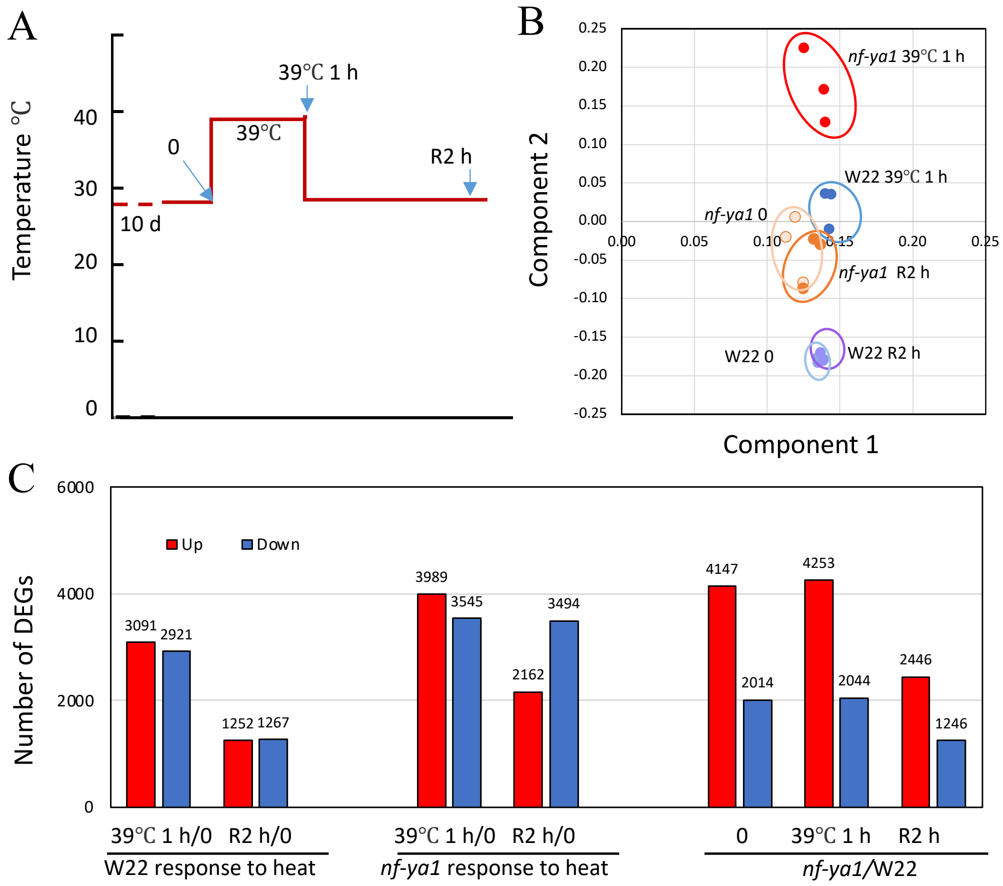

Figure S3 Principal component analysis (PCA) and Differentially expressed genes (DEGs) analysis.

(A) Schematic of the heat stress treatment of the RNA samples. Ten-day-old maize seedlings were subjected to 39 °C moderate heat stress for 1 h and then allowed to recover. Leaves samples from three biological replicates were collected at 0, 1, and 2 h after recovery (R2h) for RNAseq analysis. **(B)** PCA of data from the RNA-seq analysis. Different colors and circles indicate different samples used. **(C)** DEGs in the comparison of *nf-ya1* to W22, and their response to heat stress treatment. The number on the top of the bar was the number of the DEGs identified in the comparison. The criteria of FDR ≤ 0.01 and the absolute value of log_2Ratio ≥ 1 were used as thresholds to judge the significance of differences in gene expression.

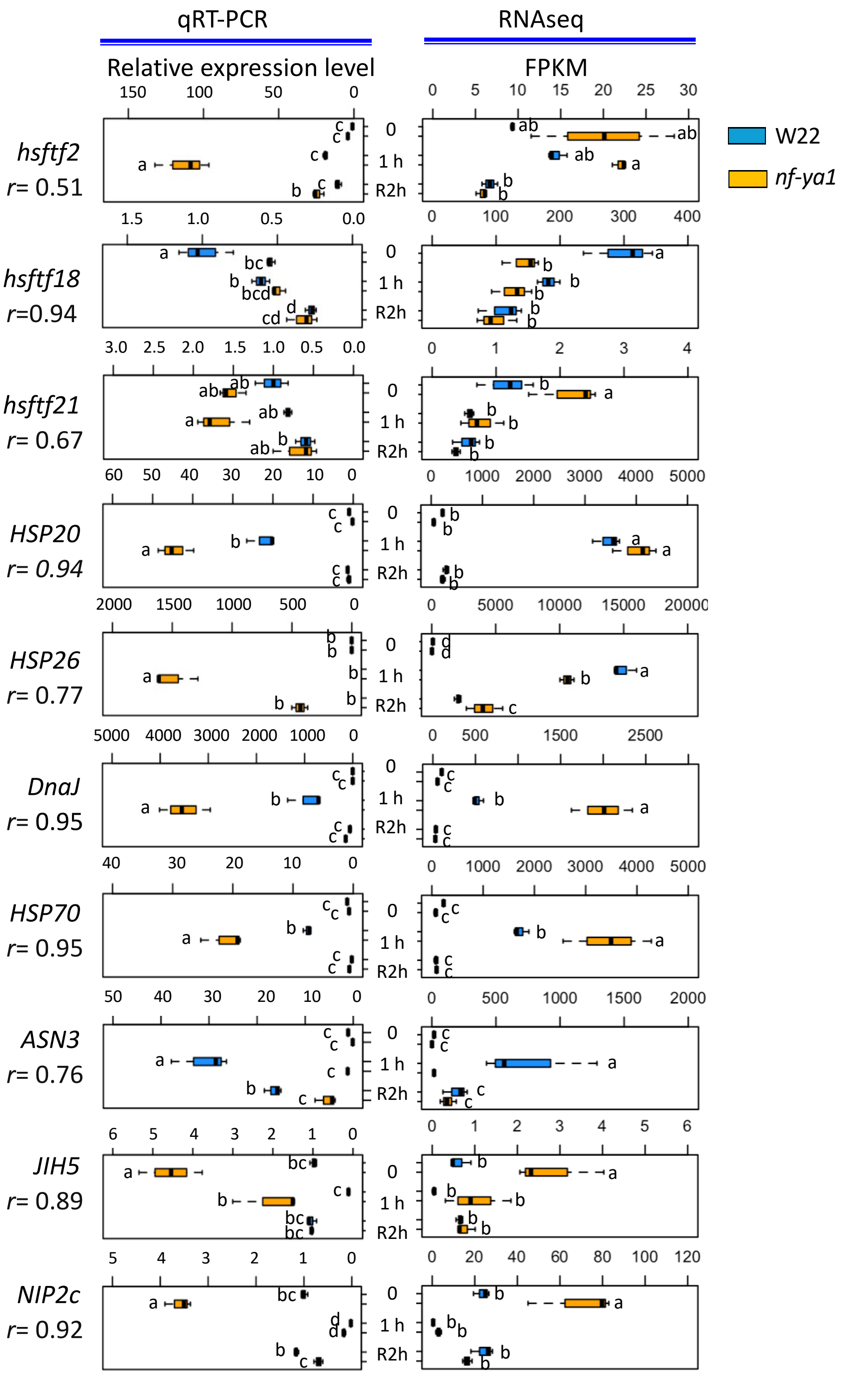

Figure S4 qRT-PCR analysis of genes in the leaves of W22 and *nf-ya1* to validate the accuracy of RNA-seq.

Ten candidates with significantly expressed different comparisons were selected to validate the transcriptome analysis. For each gene, the right panel was the gene expression changes from qRT-PCR and the right panel was the gene expression changes from RNA-seq analysis. Data analysis was the same as described in **Figure S1**. The Pearson correlation coefficient (r) between the fold changes obtained from qRT-PCR and RNAseq was calculated. Values represent the mean of three replicates ± SD. Different letters denote statistical significance with P<0.05 using ANOVA and TukeyHSD test.

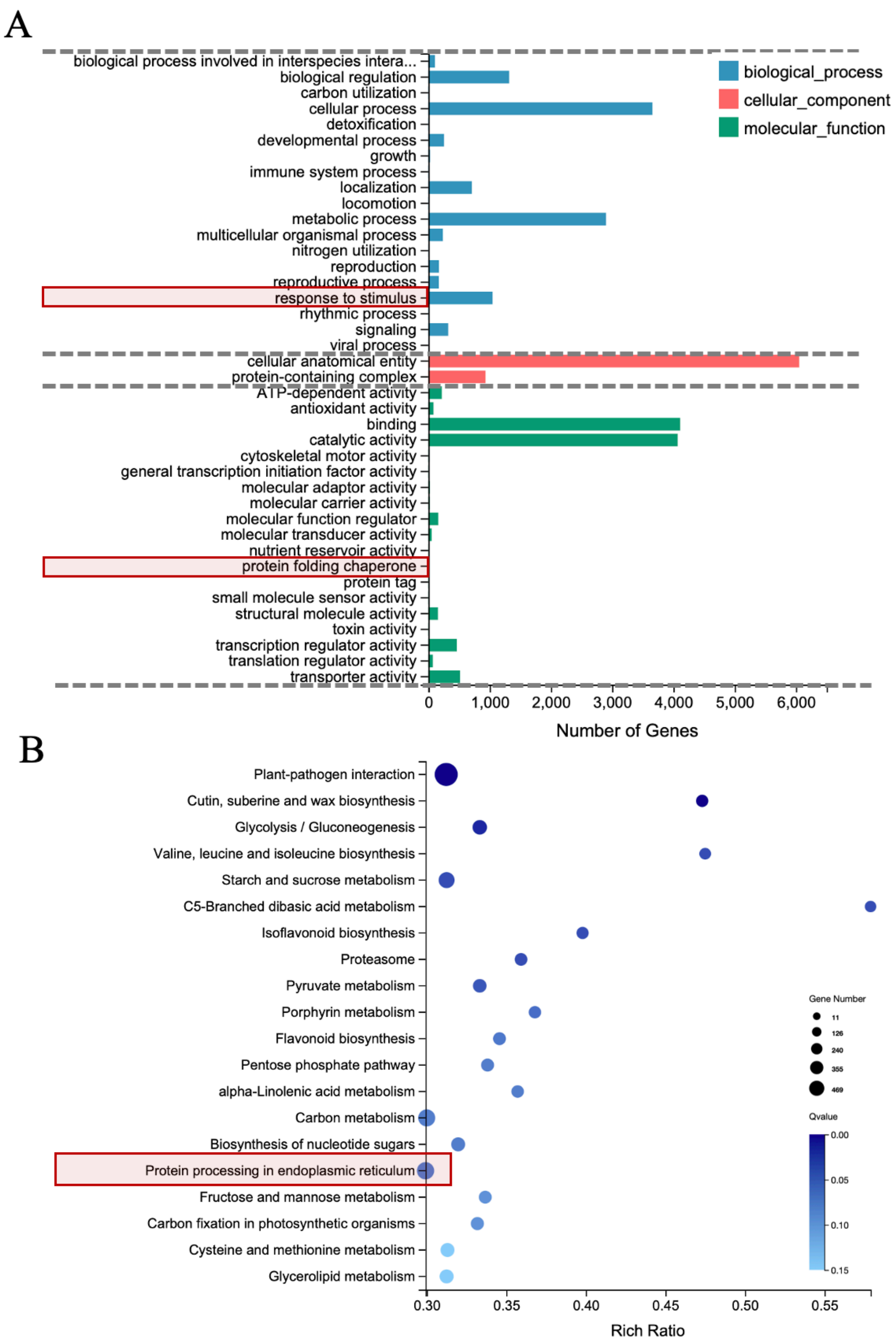

Figure S5 GO and pathways enrichment of the DEGs in comparing *nf-ya1* to W22 under normal conditions

(A) Significantly enriched GO terms of DEGs in comparing *nf-ya1* to W22 under normal conditions. The FDR value< 0.01 and absolute log2 ratio > 1 were used as cutoffs for the DEGs. The top enriched GO terms in biological process, molecular function, and cellular component were plotted. **(B)** pathways enrichment analysis of the DEGs in comparing *nf-ya1* to W22 under normal conditions. Gene number, q-value, and enriched fold were shown in the bubble plot. The red boxes indicate the GO terms or pathways highly related to ZmNF-YA1. The criteria of DEGs are described in **Figure S3**.

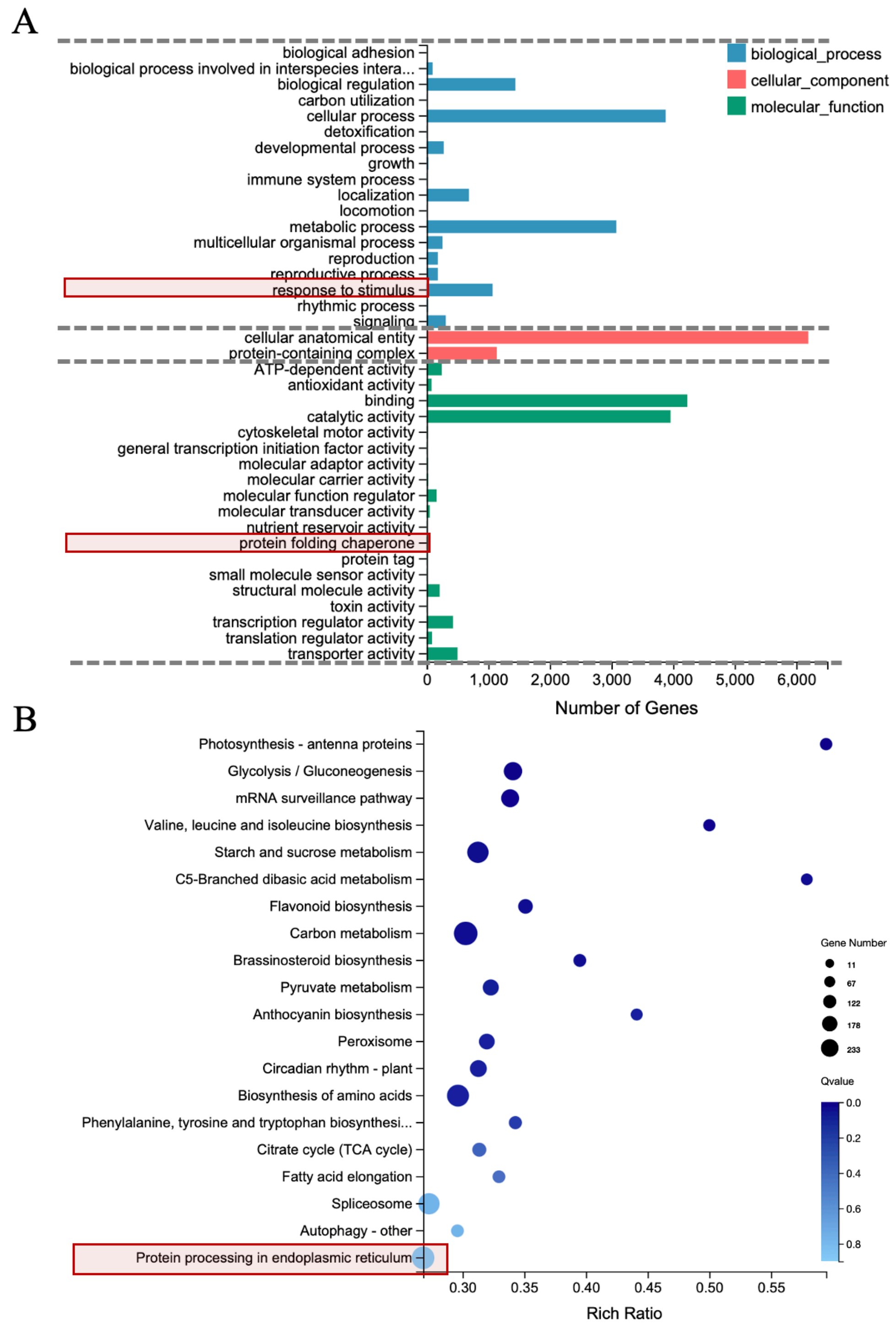

Figure S6 GO and pathways enrichment of the DEGs in comparing *nf-ya1* to W22 under heat stress conditions (39°C, 1 h)

(A) Significantly enriched GO terms of DEGs in comparing *nf-ya1* to W22 under heat stress conditions (39°C, 1 h). The FDR value< 0.01 and absolute log2 ratio > 1 were used as cutoffs for the DEGs. The top enriched GO terms in biological process, molecular function, and cellular component were plotted. **(B)** pathways enrichment analysis of the DEGs in comparing *nf-ya1* to W22 under heat stress conditions (39°C, 1 h). Gene number, q-value, and enriched fold were shown in the bubble plot. The red boxes indicate the GO terms or pathways highly related to ZmNF-YA1. The criteria of DEGs are described in **Figure S3**.

A *nf-ya1*/W22 normal condition

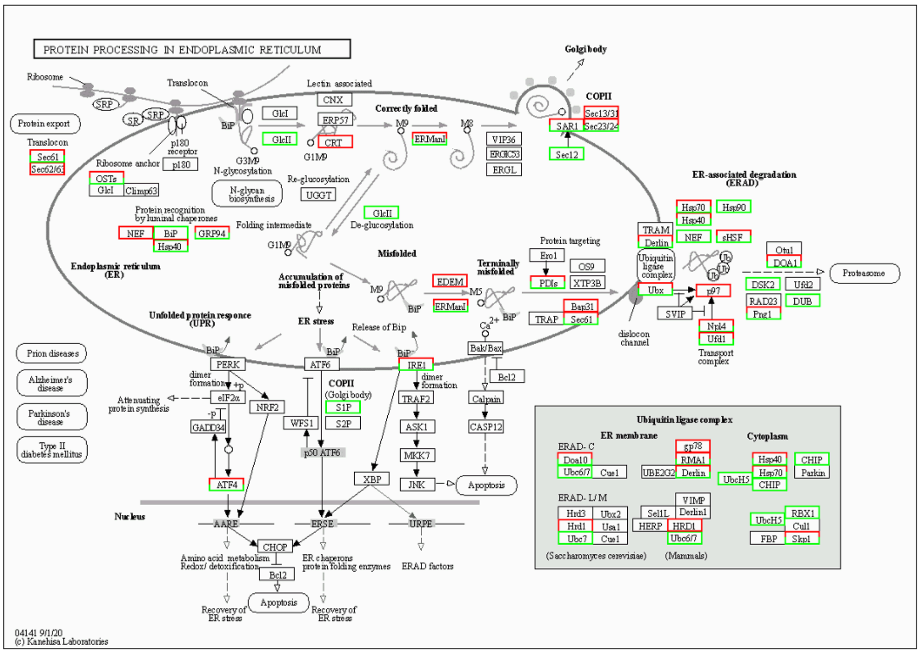

B *nf-ya1*/W22 heat stress

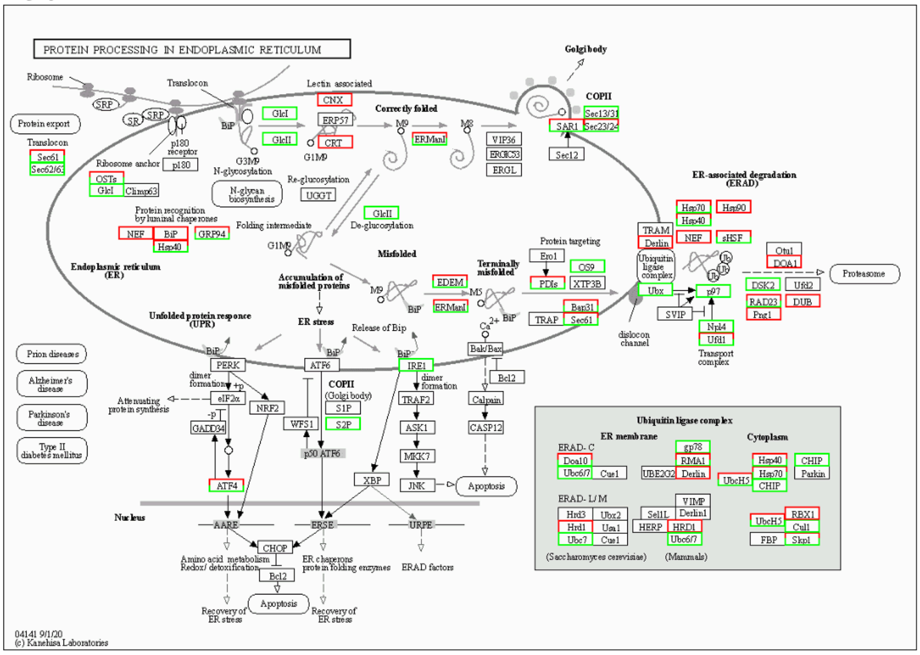

Figure S7 The DEGs involved in protein processing in Endoplasmic reticulum

The DEGs involved in protein processing in Endoplasmic reticulum in comparing *nf-ya1* to W22 under normal conditions **(A)** and heat stress treatment **(B)**. pathways modified according to KEGG. The DEGs identified in different comparisons were mapped into KEGG and the selected protein processing in the Endoplasmic reticulum in **Figures S5** and **S6** were plotted. The green boxes indicate the downregulated genes in the comparisons and the red boxes indicate the upregulated genes. The boxes with both red and green indicate that some of the genes in that group members (enzymes coding by multiple genes) were upregulated, and some were downregulated.

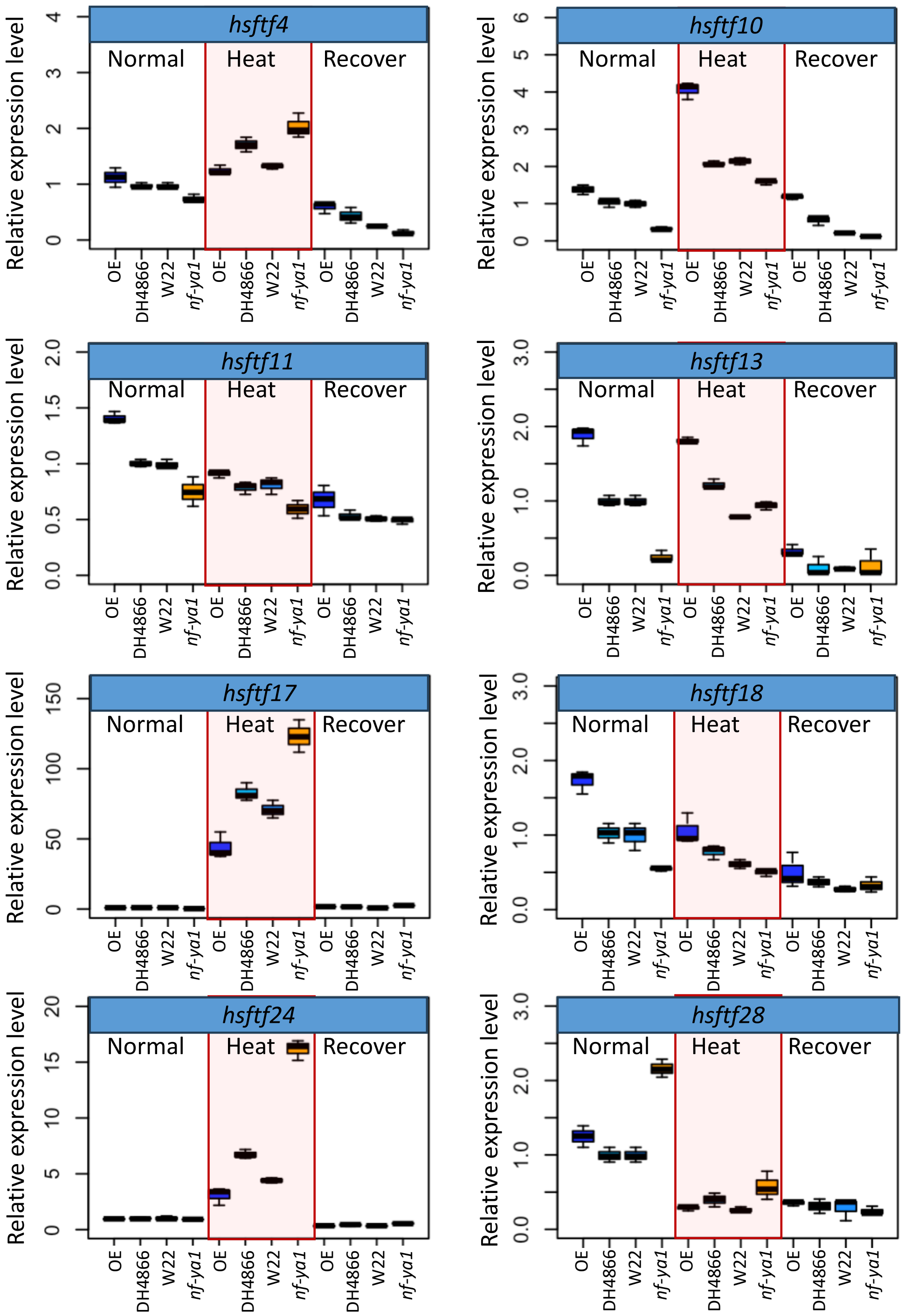

Figure S8 Expression analysis of the heat shock transcription factors in *nf-ya1*, *ZmNF-YA1* overexpression lines (OE), and their WT in response to heat stress

Eight heat shock transcription factors with significantly expressed different comparisons were selected to validate the transcriptome analysis. The transgene donor inbred line DH4866, *ZmNF-YA1* overexpression line OE1179 (OE), *nf-ya1* mutant, and its control W22 were used. Ten-day-old maize seedlings were subjected to 39 °C moderate heat stress for 1 h and then allowed to recover. Leaves samples from three biological replicates were collected at 0, 1, and 2 h after recovery (R2h) for qRT-PCR analysis. Data analysis was the same as described in **Figure S1**. Values represent the mean of three replicates ± SD.

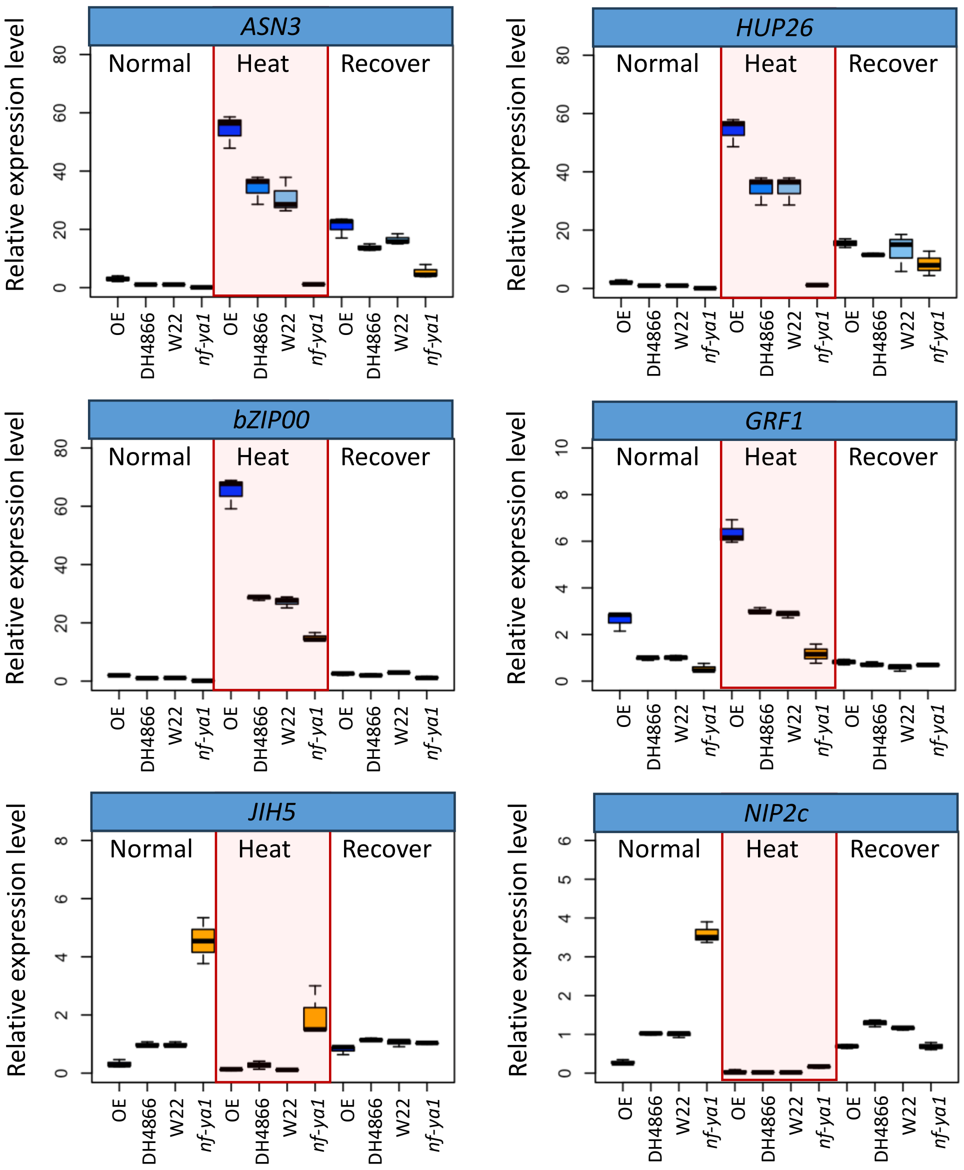

Figure S9 Expression analysis of *ASN3*, *HUP26*, *bZIP100*, *GRF1*, *JIH5,* and *NIP2c* in *nf-ya1*, *ZmNF-YA1* overexpression lines (OE), and their WT in response to heat stress

Six genes in Figure 7 (*ASN3*, *HUP26*, *bZIP100*, *GRF1*, *JIH5,* and *NIP2c*) with significantly expressed different comparisons were selected to validate the transcriptome analysis. The transgene donor inbred line DH4866, *ZmNF-YA1* overexpression line OE1179, *nf-ya1* mutant, and its control W22 were used. Ten-day-old maize seedlings were subjected to 39 °C moderate heat stress for 1 h and then allowed to recover. Leaves samples from three biological replicates were collected at 0, 1, and 2 h after recovery (R2h) for qRT-PCR analysis. Data analysis was the same as described in **Figure S1**. Values represent the mean of three replicates ± SD.
